# Experimental Investigation on Machinability of Polypropylene Reinforced with Miscanthus Fibers and Biochar

**DOI:** 10.3390/ma13051181

**Published:** 2020-03-06

**Authors:** Dinh Son Tran, Victor Songmene, Anh Dung Ngo, Jules Kouam, Arturo Rodriguez-Uribe, Manjusri Misra, Amar Kumar Mohanty

**Affiliations:** 1 Department of Mechanical Engineering, École de Technologie Supérieure (ÉTS), Montreal, QC H3C 1K3, Canada; dsontran@gmail.com (D.S.T.); AnhDung.Ngo@etsmtl.ca (A.D.N.); jules.kouam@etsmtl.ca (J.K.); 2 Department of Mechanical Engineering, The University of Danang - University of Science and Technology, 54, Nguyen Luong Bang Street, Danang 550000, Vietnam; 3 Bioproducts Discovery and Development Centre, Department of Plan Agriculture, Crop Science Building, University of Guelph, Guelph, ON N1G 2W1, Canada; rarturo@uoguelph.ca (A.R.-U.); mmisra@uoguelph.ca (M.M.); mohanty@uoguelph.ca (A.K.M.)

**Keywords:** Hybrid biocomposite, drilling, machinability, thrust force, specific cutting energy, surface roughness, fine particle, ultrafine particle

## Abstract

The machinability of composite materials depends on reinforcements, matrix properties, cutting parameters, and on the cutting tool used (material, coating, and geometry). For new composites, experimental studies must be performed in order to understand their machinability, and thereby help manufacturers establishing appropriate cutting data. In this study, investigations are conducted to analyze the effects of cutting parameters and drill bit diameter on the thrust force, surface roughness, specific cutting energy, and dust emission during dry drilling of a new hybrid biocomposite consisting of polypropylene reinforced with miscanthus fibers and biochar. A full factorial design was used for the experimental design. It was found that the feed rate, the spindle speed, and the drill bit diameter have significant effects on the thrust force, the surface roughness, and the specific cutting energy. The effects of the machining parameters and the drill bit diameter on ultrafine particles emitted were not statistically significant, while the feed rate and drill bit diameter had significant effects on fine particle emission.

## 1. Introduction

Natural fiber reinforced polymer (NFRP) composites are used in technical area such as construction, automotive parts, and sports’ goods due to their many advantages, for instance, low density, high specific strength and stiffness, low hazard manufacturing process, and low cost [1]. Manufacturers are expected to use NFRPs as replacement of synthetic fiber reinforced polymer (SFRP) composites when producing automobile parts in order to minimize environmental impacts and reduce vehicle weight [2]. Products made with composite materials need to be machined (for example, drilled) in order to obtain the final dimensions or for assembly purposes. Conventional drilling is a popular hole making method, and an indispensable process for creating holes for parts assembly [3].

Many publications have covered the machining of SFRPs, while little research work has been done on the machining of NFRPs. Results relating to the machinability of SFRPs cannot be directly applied to NFRPs because of the differences in the mechanical properties of these two composites [4]. Debnath et al. [5] investigated the drilling behavior of nettle/polypropylene composite with various drill bit geometries. They found that a specific drill bit geometry is only suitable for cutting one material, but is not appropriate for cutting others. Consequently, studying the cutting mechanism is necessary so that each material’s machinability can be fully understood. Bajpai and Singh [6] studied the machinability of sisal/polypropylene composite during the drilling process. They observed that the thrust force created with the standard drill tool is significantly bigger than that obtained with the trepanning tool. Maleki et al. [7] carried out an investigation on the machining of woven jute fabric reinforced polymer composite with different drill tool materials. They [7] showed that the delamination factor and thrust force are lower with the HSS drill compared to those obtained with carbide drills (CoroDrill 854 and CoroDrill 856). The surface roughness generated with the CoroDrill 856 was higher than that made with the HSS tool and the carbide CoroDrill 854. They recommended using the HSS twist drill when drilling jute fiber reinforced polymer composites [7]. 

The type of fibers used in composite materials has a great influence on the machinability of NFRPs since the tool used will cut both the fibers and the matrix. Therefore, in addition to machining cutting parameters, the properties of the fibers must be taken into account when selecting the cutting tool [4]. Ismail et al. [8] investigated the drilling of sustainable and conventional composites (HFRP—hemp fiber reinforced polycaprolactone, and CFRP—carbon fiber reinforced polymer). They observed that cutting parameters can significantly affect the damage sustained by both composites. The thrust force, the roughness, and delamination increased with feed rate increase for these materials. Their results also showed that CFRP presents a lower surface roughness value, and that HFRP exhibits a smaller delamination factor during drilling process in the same cutting conditions. Abilash and Sivapragash [9] studied the influence of cutting condition on delamination during drilling of polyester composite reinforced with bamboo fiber. They found that the feed rate and the drill bit diameter play a larger role in delamination failure. Venkateshwaran and ElayaPerumal [10] presented that an increase in the feed rate and the spindle speed contributed to the rise of the delamination factor during drilling of epoxy composite reinforced with banana fibers. Chegdani and Mansori [11] found that the use of coated tools generated an increase in specific cutting energy in the drilling of bidirectional flax fiber reinforced polypropylene. The study [12] explored the machinability of two types of green composites (Sisal/PLA and Grewia Optiva/PLA). They noticed that drilling with a twist drill and a parabolic drill produces a better surface quality and a lower thrust force than does the Jo drill tool.

Debnath et al. [13] investigated the drilling of sisal reinforced epoxy and polypropylene composites. They found that the machinability of sisal composite depends not only on the tool geometry, but also on the matrix material. The thrust force and torque generated are lower for the parabolic drill than for the step and four-facet drills. Higher torque values were observed for sisal/epoxy than for to sisal/polypropylene at the same cutting condition. No delamination was observed for both materials. Palanikumar and Valarmathi [14] studied the drilling of MDF (Medium Density Fiberboard, which is a wood-based composite) and discovered that the thrust force increased with the feed rate. Prakash et al. [15] observed that the roughness increased with the drill bit diameter and feed rate during drilling of MDF. Szwajka et al. [16] noted that the tool coating type and the feed rate significantly influenced the thrust force and the roughness when machining MDF. 

Hybrid composite materials are made from a common matrix reinforced with more than one type of reinforcements. Fu et al. [17] defined hybrid composites as having two or more reinforcing materials mixed in a common matrix. Recently, some studies have looked at the machinability of hybrid composites. Jayabal et al. [18] investigated the machinability during drilling of glass-coir/polyester hybrid composite. They found that the feed rate has a more significant effect on the thrust force than the drill diameter and spindle speed do. Navaneethakrishnan and Athijayamani [19] observed the effect of cutting conditions on the cutting force during drilling of vinylester reinforced with sisal fiber and coconut shell powder. They discovered the drill point angle and the feed rate have the significant impact on thrust force. Increases in the point angle and the feed rate contribute to increases in the thrust force. Vinayagamoorthy [20] observed that a decrease in the spindle speed and/or an increase in the feed rate lead to the rise of the thrust force and the roughness during drilling of polyester composite reinforced with jute and steel fiber. They also discovered that the thrust force increases with a drill diameter and drill point angle increase.

The dust generated during machining of metallic materials has been studied by several researchers [21,22,23,24,25,26,27,28,29,30,31], who observed that the relationship of the dust emission with cutting conditions such as the workpiece material, tools, and cutting parameters [21,28,31]. Small size dust emitted during machining has a significant impact on the environment and on the health of machine operators [22,29]. Djebarra et al. [24] observed that during machining, the greatest proportion of dust generated is less than 2.5 microns, for different workpiece materials at different cutting conditions. The quantity of particles emitted is a function of the spindle speed and the feed rate [22,26]. Kouam et al. [27] found that friction has a great impact on dust formation. Zaghbani et al. [25] noted that the deformation conditions in the chip formation zone greatly influence the dust generated, while the cutting conditions do not significantly affect the nanoparticle generation rate. The result from [26] indicates that the tool coating materials influence the dust generated during the machining of aluminum alloys. The study [29] investigated the effect of the initial temperature of the workpiece material on fine dust emission during dry drilling and found that the initial workpiece temperature greatly affects the dust generation. For pre-cooled workpiece materials, the equivalent generation was low. 

To date, only a few studies have focused on dust generated during machining of composites. Marani et al. [32] found that the cutting parameters and the microstructure of the workpiece material directly affect the dust generated during metal matrix composite milling. Songmene et al. [33] found that fine dust generation is significantly reduced by using water MQL (Minimum Quantity Lubrication) during polishing of granite, while ultrafine particle generation is insensitive to water use. Kremer and Mansori [34] observed that a smooth coating tool generates more dust than a rough one during machining of metal matrix composite. The study Kremer and Mansori [35] showed that the dust created during cutting of metal matrix composite is affected differently depending on the tool type. Haddad et al. [36] revealed that dust emission is a function of cutting parameters and the tool geometry during the trimming of polymer composite reinforced with carbon fibers. 

The aim of this study is to investigate the effects of machining conditions on the machining process performance indicators: Specific cutting energy, thrust force, surface roughness, and fine dust and ultrafine dust emission during dry drilling of a new hybrid biocomposite material made of miscanthus fibers and biochar reinforced polypropylene. 

## 2. Experimental Setup

### 2.1. Workpiece Material

The hybrid biocomposite material used consists of a matrix (polypropylene (PP)/polyolefin elastomer (POE)) randomly reinforced with biochar and chopped miscanthus fiber, and mixed with a MAPP (Maleic Anhydride grafted Polypropylene) compatibilizer. The composition of the hybrid biocomposite is presented in Table 1.

**Figure 1:**
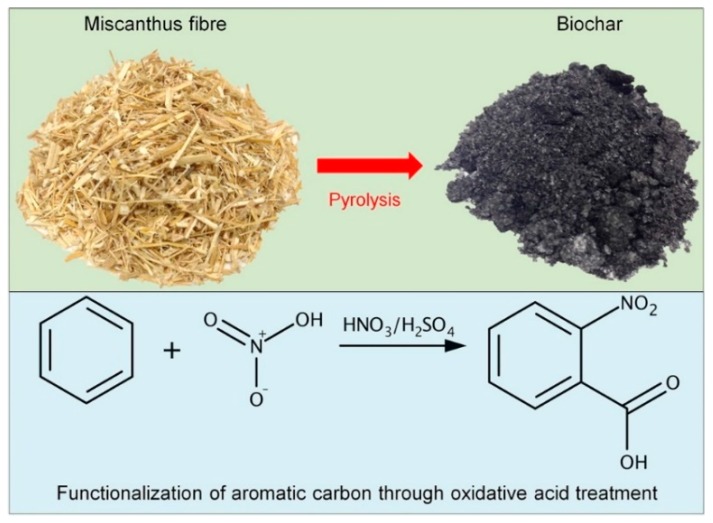
Fabrication of biochar [37].

PP (trade name, 1350N) is produced by Pinnacle Polymers LLC, LA, USA. POE (trade name, Engage 7487); MAPP (trade name, Fusabond 613). The biochar is the result of the pyrolysis operation of natural miscanthus fibers (average length of 4 mm) invented in Southern Ontario, Canada. Competitive Green Technologies supplied four millimeters long miscanthus fibers that were used for the manufacturing of the hybrid composite. Figure 1 describes the miscanthus fibers and biochar that were made from miscanthus fibers by pyrolysis [37]. The mechanical properties of hybrid biocomposite are described in Table 2 [38].

The hybrid biocomposite material was produced through the press molding process, using a French Press USA machine, TMP, Model EHV, max tonnage 57 tons. The following steps represent the process for producing compression-molded materials. Firstly, the mold platens are preheated to 180 °C for 30 min. Then, the material is loaded into the press and pre-heated with closed platens, but without raising the pressure in order to melt material before pressing. This operation takes about 10 min. Then vacuuming is conducted to degas for 3 min, and platens are closed at a pressure of 2 tons for 10 min. Finally, the platens are cooled to below 50 °C and then removed from the press. 

The hybrid biocomposite material was developed in 2017 by the University of Guelph Bioproducts Discovery and Development Center for internal automobile parts production. The result obtained from [38] showed this hybrid biocomposite exhibited better stiffness than the matrix (PP/POE). The Young’s modulus of the matrix was increased by 70% as reinforced with biochar (15%wt) and miscanthus (15%wt). Hybrid biocomposite has higher tensile and flexural strength than commercial Talc/PP composite (RTP132UV). As a result, hybrid biocomposite is considered the most likely material to replace the commercial Talc/PP composite. 

### 2.2. Experimental Procedure

The dry drilling of hybrid biocomposite was conducted on a 3-axis CNC machine-tool (HURON—K2X10) with the following main characteristics: Maximum power, 50 KW; and spindle speed, 28,000 rpm. Standard HSS twist drill bits (6 mm, 8 mm, 10 mm diameters) were used to drill holes on the workpiece. The cutting parameters were selected based on the tool manufacturer’s catalogue and literature.

The workpiece sample (300 mm × 120 mm × 5 mm) was screwed to an aluminum back plate support. The support (300 mm × 120 mm × 30 mm) had 80 drilled holes (12 mm diameter). The subsystem (workpiece and back plate support) was placed and tightened onto the dynamometer with screws. The responses measured and analyzed were the drilling force, the surface roughness, and the particle emission. The drilling forces were measured using a dynamometer (type Kistler 9255B) clamped on the machine table and connected to the charge amplifiers (type Kistler 5010) (Kistler Instrument Corporation, New York, NY, USA) that generated output signals, which were transmitted to a data translation card (type DT 9836, Data Translation Inc., Marlborough, MA, USA) and then connected to a personal computer.

**Figure 2:**
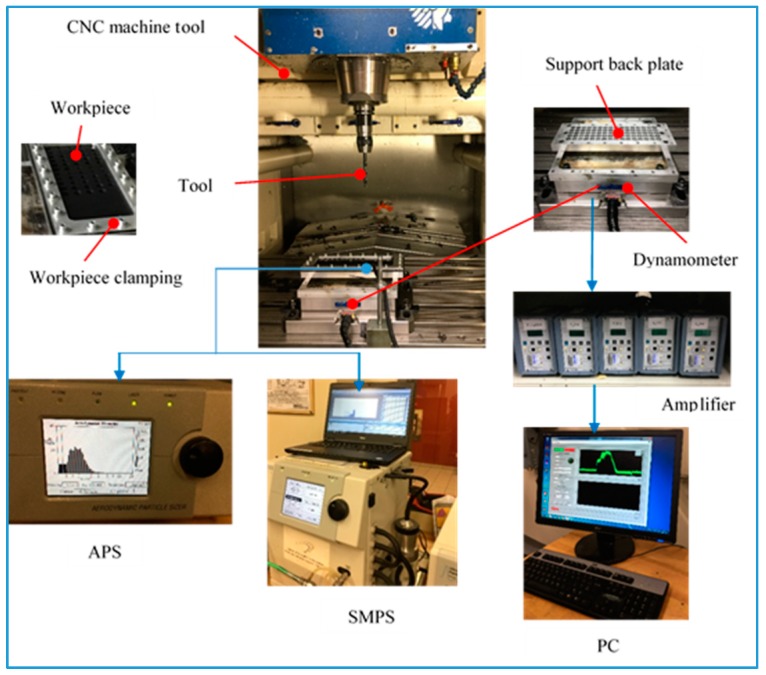
The experimental devices for machining and measurement system.

A Scanning Mobility Particle Sizer (SMPS, model #3080, TSI Inc., Shoreview, MN, USA) equipped with a nano DMA (Differential Mobility Analyzer) was used to measure ultrafine particles generated with sizes ranging from 7 nm to 100 nm during drilling. An Aerodynamic Particle Sizer (APS, model 3321, TSI Inc., Shoreview, MN, USA) was used for measuring of fine particles, with diameter ranging from 0.5 to 10 μm. For both pieces of equipment, the dust samples were sucked by a pump (1.5 L/min) through a suction tube, with the end of a tube placed near the machining area. The suction tube was connected to the dust measurement system, which consisted of APS and SMPS. The experimental scheme is illustrated in Figure 2.

The roughness profilometer (Mitutoyo, model SJ–410) (Mitutoyo America Corporation, Aurora, IL, USA) was used to measure the surface roughness. This equipment was connected to a computer with the help of the SURFPAK–SJ software for recording and analyzing the roughness data. The measurements of the surface roughness of a drilled hole were carried out in the feed direction, and were repeated three times for each tested condition. 

The experiments were based on a full factorial design, with 3 input parameters at 3 levels. In order to obtain reliable and accurate results, each test was repeated three times, and the mean of the measured values was selected for analysis. Table 3 summarizes the factors investigated and their respective levels.

## 3. Results and Discussion

### 3.1. Thrust Force

The thrust force was calculated as the average value from the thrust force signals when the main cutting edges and the chisel edge of the drill were in full contact with the workpiece. Table 4 presents the analysis of variance for the thrust force. The effect of the main factors and their interaction on the drilling process was analyzed by ANOVA. The main objective of ANOVA is to apply the statistical method to understand the effect of the individual factors. It is found that four factors have *p*-values smaller than 0.05, which are statistically significant at the 95% confidence interval. The feed rate (f) has the highest effect on the thrust force, followed by the spindle speed (s), the drill bit diameter (d) and the interaction sd. It is also shown that the interaction fs and the interaction fd are not statistically significant for the thrust force.

The factors with statistically significant effects were selected to develop the experimental prediction model for the thrust force using regression analysis. The thrust force was related to the machining parameters as follows:

(1) $$F_{t}=\;42.82\;+\;225.11\;\times \;f\;-\;0.031\;\times \;s\;-\;2.68\;\times \;d\;+\;5.95{\;\times \;10}^{-3}\;\times \;s\;\times \;d$$

where F_t_ is the thrust force (N), and f, s, and d denote the feed rate (mm/rev), the spindle speed (rpm), and the drill bit diameter (mm), respectively. The coefficient of correlation obtained from the ANOVA, R^2^ was 89.23% and the R^2^-adjusted was 87.27%. These coefficients indicate that the model is adequate for prediction of thrust force.

**Figure 3:**
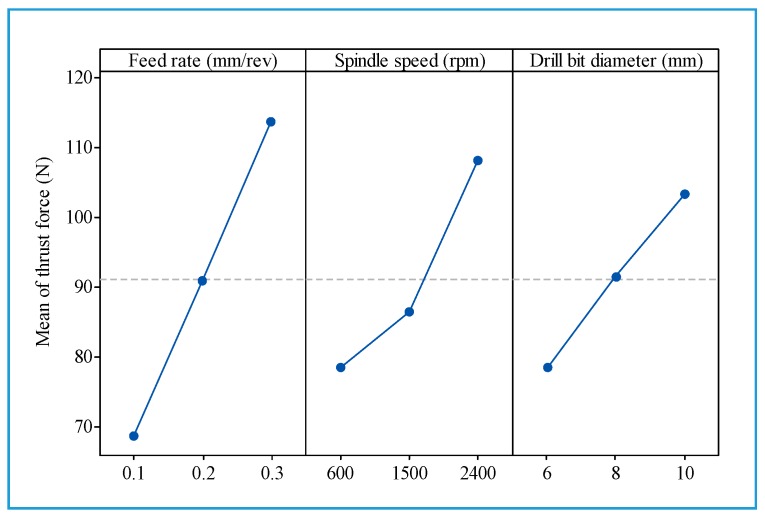
The main effects for thrust force.

Figure 3 shows the main effects of the tested factors on thrust force. It can be seen that the feed rate has a larger effect on the thrust force than do the spindle speed and drill bit diameter. This is in agreement with other observations [18]. Generally, the thrust force increased with the drill diameter, the feed rate, and the spindle speed.

**Figure 4:**
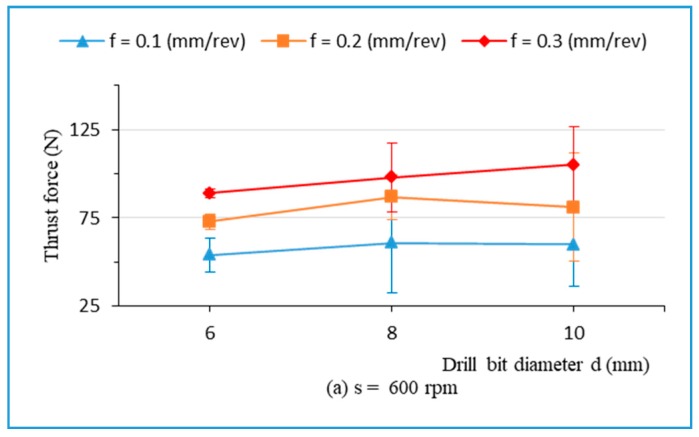

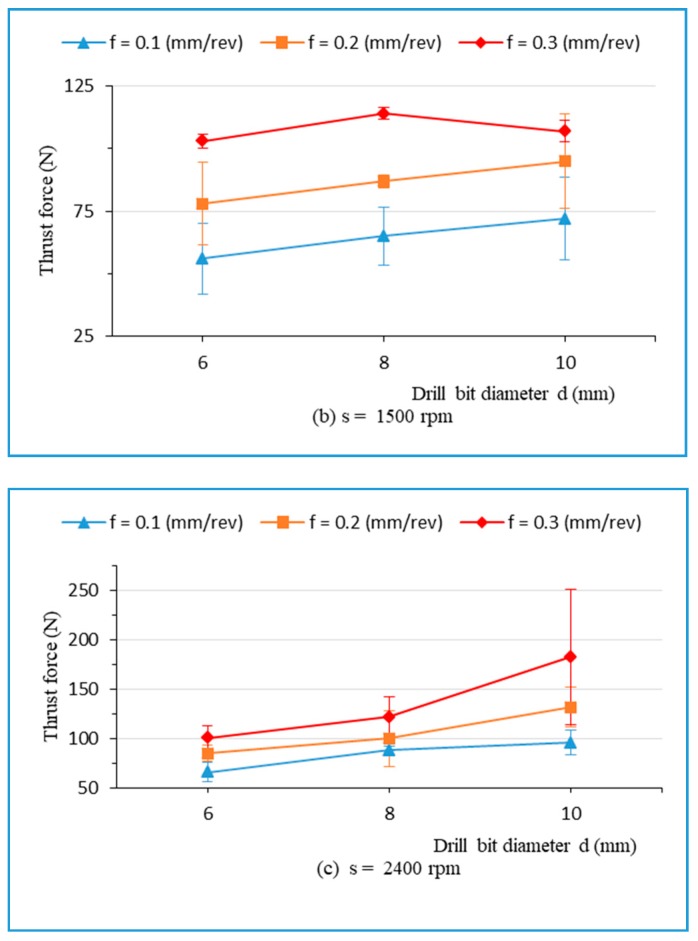
Thrust force related to drill bit diameter during drilling of hybrid biocomposite with different feed rates, at spindle speeds: (**a**) s = 600 rpm, (**b**) s = 1500 rpm, and (**c**) s = 2400 rpm.

In Figure 4, it can be seen that the rise of thrust force follows an increase in feed rate. It can be interpreted that an increase in feed rate contributed to the larger of the cross-sectional area of the uncut chip. This led to an increase in the resistance to chip formation, and subsequently, to the higher thrust force value. This result is consistent with other studies [5,7,12,13,14,39,40]. It also indicates that the thrust force increases rapidly with feed rate increase, especially at the highest spindle speed of 2400 rpm, with a drill of 10 mm (Figure 4c).

Figure 3 and Figure 4c show that an increase of the thrust force results from the use of larger drill’s diameter. This can be explained by the fact that an increase on drill bit diameter leads to an increase in the cross-sectional area of the uncut chip and the chisel edge width. Therefore, higher cutting forces are required. Furthermore, an increase in the drill’s diameter makes the contact area between the workpiece material and drill bit larger, which results in increased friction, and thus thrust force increases [20]. This is in agreement with the results obtained from other studies [20,40]. 

Figure 4 also depicts the interaction between the feed rate, the spindle speed, and the drill bit diameter on thrust force. It presents the thrust force in relation to the drill bit diameter with different feed rates and spindle speeds: 600 rpm (Figure 4a), 1500 rpm (Figure 4b), and 2400 rpm (Figure 4c). It is seen that the thrust force increases slightly with drill bit diameters ranging from 6 mm to 8 mm at different spindle speeds. Nevertheless, the thrust force has an unclear trend, with drill bit diameters ranging from 8 mm to 10 mm due to the interaction between the spindle speed and the drill bit diameter (Figure 4a–c). In general, the thrust force rose slowly with an increase in the drill bit diameter at spindle speeds ranging from 600 rpm to 1500 rpm. However, the thrust force increased significantly with drill’s diameters at s = 2400 rpm.

**Figure 5:**
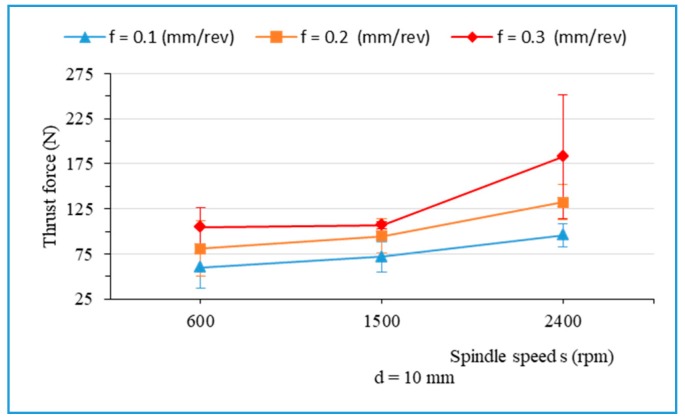
Thrust force versus the spindle speed when drilling hybrid biocomposite using drill bit diameter of 10 mm with various feed rates.

From Figure 3 and Figure 4, it can be found that the thrust force increases with the spindle speed increase. Figure 5 indicates that the thrust force rises slowly with the spindle speed (in the range of 600 rpm to 1500 rpm), and then increases significantly as the spindle speed rises up to 2400 rpm. This can be explained as follows:

The linear feed rate in mm/min, v_f_, is expressed by v_f_ = f * s, where s is the spindle speed in revolution per minute (rev/min), and f is the feed rate in millimeter per revolution (mm/rev). It showed that the spindle speed increase leads to an increase in the linear feed rate v_f_ (mm/min) due to fact that the feed rate is an independent parameter. Consequently, the spindle speed increase results in an increase in the thrust force. This is similar to the results of other investigations, with an increase in the linear feed rate (mm/min) leading to an increase in thrust force [14,39,40]. The maximum thrust force value was obtained at the highest value of input parameters (f = 0.3 mm/rev, s = 2400 rpm, and d = 10 mm).

### 3.2. Specific Cutting Energy for Thrust Force

The specific cutting energy (also called the specific cutting pressure) is defined as the cutting force per unit area of the uncut chip. During the drilling process with the standard twist drill bit, K_t_ denotes the specific cutting energy for the thrust force (N/mm^2^), and is calculated as in the following equation [3]:

(2) $${\;K}_{t\;}\;=\;\left({2*F}_{t}\right)/\left(f*d\right)$$

where F_t_, f, and d denote the thrust force (N), the feed rate (mm/rev), and the drill bit diameter (mm), respectively. 

Table 5 presents the analysis of variance for specific cutting energy related to the thrust force. It shows that three tested factors have effects with *p*-values smaller than 0.05, which indicates that they are statistically significant at the 95% confidence level. The feed rate has the highest effect on the specific cutting energy for the thrust force. These factors were used to develop the empirical model for the prediction of the specific cutting energy for the thrust force. The result from the ANOVA shows that R^2^ is 86.65% and the adjusted R^2^ is 84.91%, which indicates that the model is suitable for prediction. The empirical model for specific cutting energy K_t_ (N/mm^2^) was obtained as follow:

(3) $$K_{t}\;=\;237.31\;-\;393.57*f\;+\;2.19{*10}^{-2}*s\;-\;7.78*d$$

where f (mm/rev) is the feed rate; s (rpm) is the spindle speed; and d (mm) is the drill diameter.

**Figure 6:**
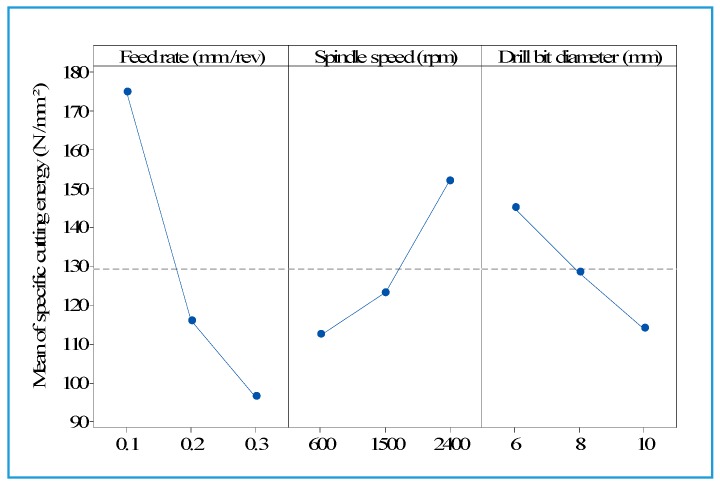
Main effects plot for specific cutting energy related to thrust force.

**Figure 7:**
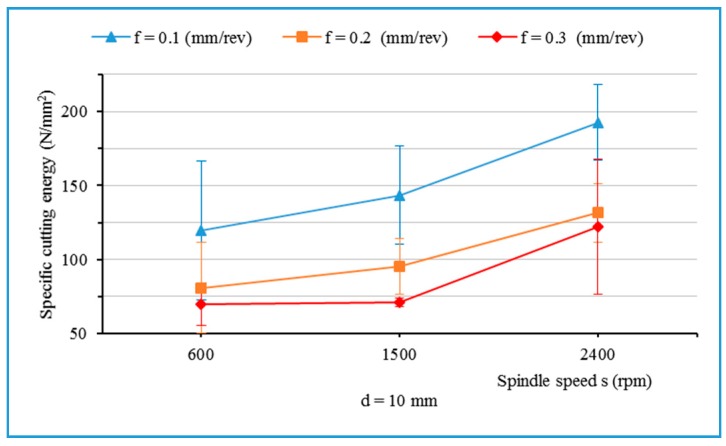
Specific cutting energy for thrust force as a function of spindle speed with different feed rates and drill bit diameter of 10 mm.

Figure 6 and Figure 7 show that the specific cutting energy for the thrust force decreased with an increase in feed rate, but increased when the spindle speed increased. This result is in agreement with the literature [11]. It is also observed that an increase in the drill’s diameter contributes to a decrease of the specific cutting energy for the thrust force (Figure 6).

### 3.3. Surface Roughness

**Figure 8:**
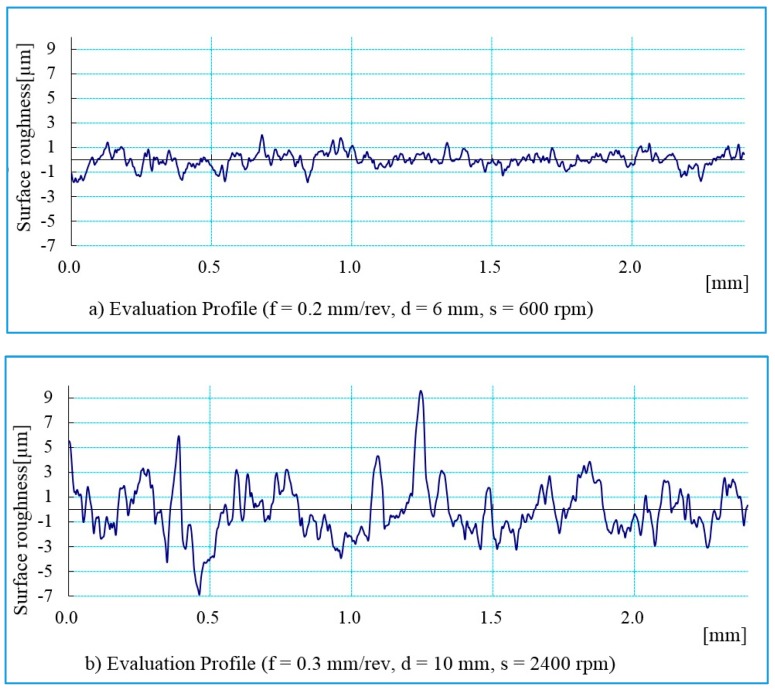
Surface roughness profile for drilled holes with different cutting conditions: (**a**) f = 0.2 mm/rev, d = 6 mm, s = 600 rpm; and (**b**) f = 0.3 mm/rev, d = 10 mm, s = 2400 rpm.

**Figure 9:**
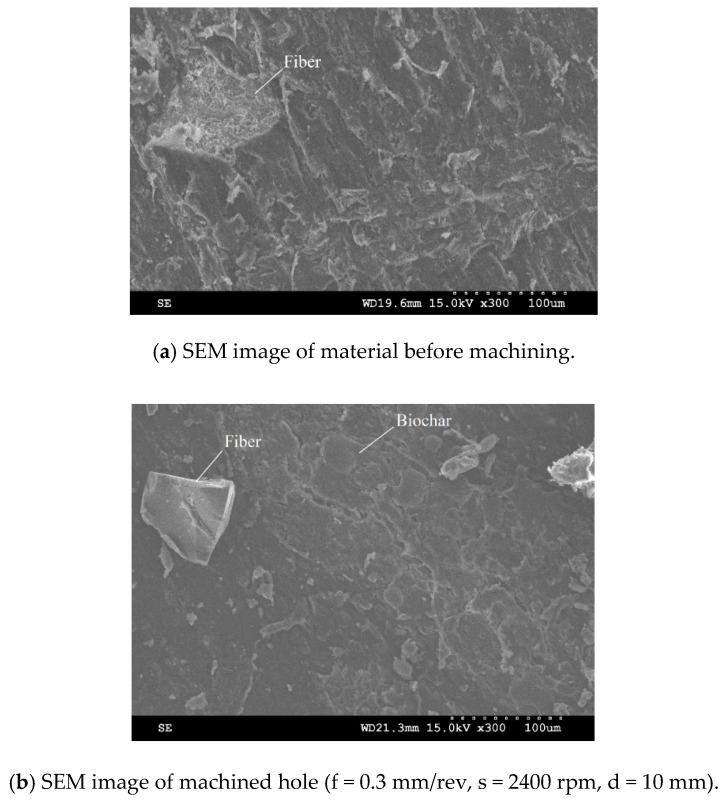
SEM images for hybrid biocomposite: (**a**) SEM image of surface before machining; (**b**) machined surface at f = 0.3 mm/rev, s = 2400 rpm, d = 10 mm.

The surface roughness is one of the criteria for determining the machined surface quality. In the present research work, the surface roughness parameters were measured in the hole in a longitudinal direction. The roughness parameters were measured three times for each hole, with a cut-off length of 0.8 mm. The mean arithmetic average roughness (R_a_) value and the maximum profile height (R_t_) obtained from the measured results were used for evaluating the effect of cutting conditions on the machined surface. Figure 8 presents the measured profile of holes drilled in the longitudinal direction, at different cutting conditions. Figure 8a shows the drilled hole with the best surface finish at cutting condition (f = 0.2 mm/rev, s = 600 rpm, d = 6 mm). The highest surface roughness value was obtained at cutting condition (f = 0.3 mm/rev, s = 2400 rpm, d = 10 mm), illustrated in Figure 8b. The morphology of the surface before machining and the machined surface with the largest surface roughness value is presented in Figure 9. The machined surface indicates that neither uncut fibers nor delamination were found. 

#### 3.3.1. The Arithmetic Average Roughness (R_a_)

Table 6 indicates that three tested factors (Feed rate, spindle speed, and drill diameter) have *p*-values smaller than 0.05. These factors are statistically significant at the 95% confidence level. It is found that the drill bit diameter has the greatest influence on the surface roughness. The interaction of each factor with others (fd, fs, and sd) have no statistical significance. The coefficient of correlation obtained was R^2^ is 85.46%, indicating the models as fitted explains 85.46% of the variability in surface roughness. The adjusted R^2^ is 83.57%, which is more suitable for comparing models with different numbers of independent variables. It showed that the model is adequate for prediction. The empirical model was obtained as follows:

(4) $$R_{a}\;=\;-\;0.22\;+\;0.48*f\;+\;16.33{*10}^{-5}*s\;+\;0.1*d$$

where f (mm/rev) is the feed rate; s (rpm) is the spindle speed; and d (mm) is the drill diameter.

**Figure 10:**
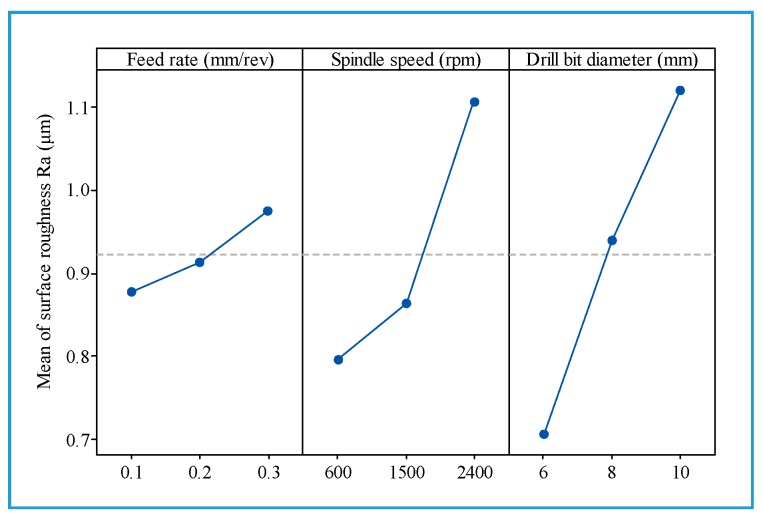
Main effects plot for the roughness (R_a_).

**Figure 11:**
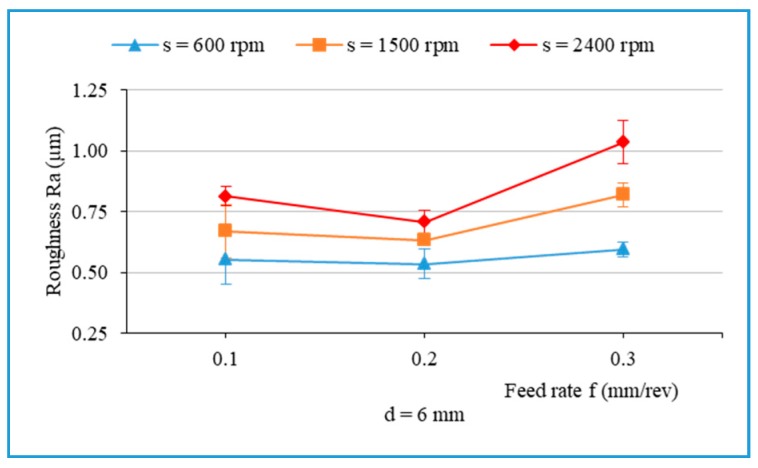
Surface roughness (R_a_) versus the feed rate when drilling with different spindle speeds, d = 6 mm.

**Figure 12:**
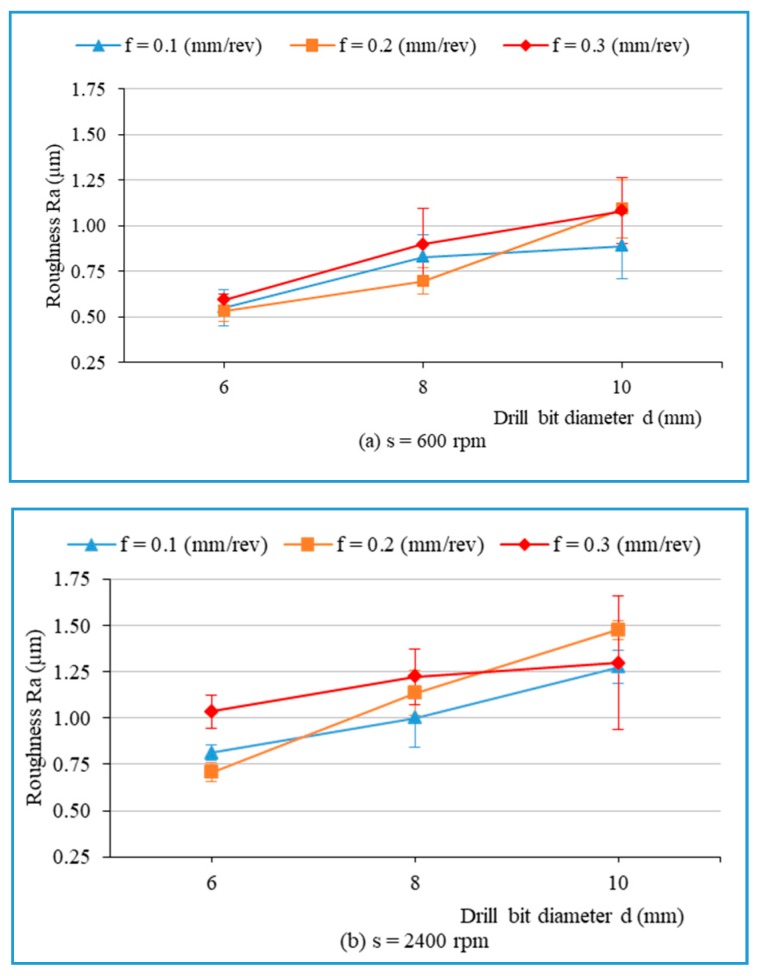
The roughness (R_a_) versus drill’s diameter with different feed rates and spindle speeds: (**a**) s = 600 rpm, (**b**) s = 2400 rpm.

Figure 10 presents the main effects on the arithmetic average roughness (R_a_). Figure 11 illustrates the influence of cutting parameters on the surface roughness when drilling hybrid biocomposite with the smallest diameter drill bit (d = 6 mm). From Figure 10 and Figure 11, it can be observed that the surface roughness increases with an increase in one of the three main factors of the drilling process in general. Figure 12 shows the influence of the drill bit diameter on surface roughness, with various cutting parameters. It is also observed that the use of the bigger drill bit diameter leads to a dramatic increase in the roughness, which is consistent with the result obtained from other investigations [15].

In Figure 11, it can be observed that R_a_ decreases slightly as the feed rate rises from 0.1 to 0.2 mm/rev, and then it experiences a dramatic increase with the rise of feed rate up to 0.3 mm/rev. It is observed that the surface roughness is more sensitive to variations of feed rate at the highest spindle speed (2400 rpm) than at the lowest spindle speed (600 rpm). It also shows the main influential trend that the surface roughness (R_a_) increases slightly with an increase in feed rate (Figure 10), which is consistent with some studies [15,39].

In Figure 10, it can be found that the spindle speed increase from 600 to 1500 rpm leads to a slight roughness increase; the roughness then increases sharply as the spindle speed rise up to 2400 rpm. This can be explained by the fact that the linear feed rate (mm/min) rises with the spindle speed increase, as mentioned above, and contributes to a reduced cutting time. This in turn results in the reduction of contact time between the drill bit and the workpiece, a reduction of the polishing effect, and a simultaneous increase in thrust force. As a result, the surface roughness increases as the spindle speed increases.

#### 3.3.2. Maximum Height of Profile (R_t_)

**Figure 13:**
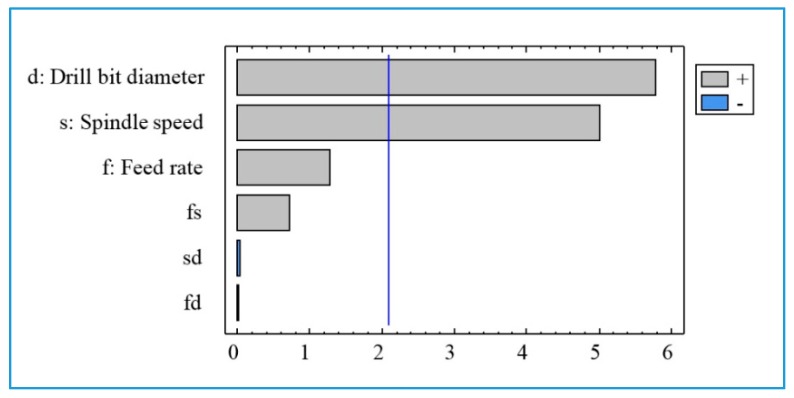
The chart of the effects for the surface roughness R_t_.

**Figure 14:**
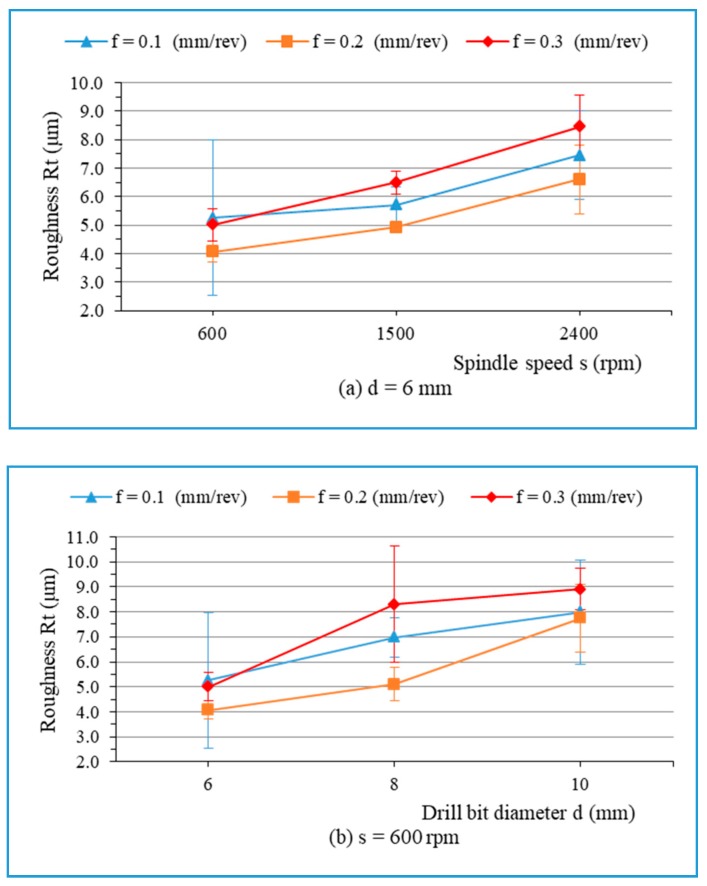
Roughness R_t_ versus the cutting conditions: (**a**) d = 6 mm; and (**b**) s = 600 rpm.

The Pareto diagram (Figure 13) illustrates the effect of the machining parameters on the roughness R_t_. It is seen that two factors (drill diameter and spindle speed) are statistically significant on the roughness R_t_. From Figure 14, it can be observed that the main influential trend of cutting parameters on the maximum height of the profile (R_t_) is similar to that of surface roughness R_a_, discussed above. The R_t_-values increase with an increase in the spindle speed (Figure 14a) and the drill bit diameter (Figure 14b). 

### 3.4. Dust Emission during Drill Process

#### 3.4.1. Fine Particle Emission 

The rotation of the drill during machining creates an airflow movement around the drill tool, which results in the dispersion of dust generated at the cutting zone. The size and quantity of the dust depend on the cutting parameters, the workpiece, and the tool material [26,31]. The aerodynamic particle sizer (APS) measures particles from 0.5 to 10 µm, and presents these results by parameters such as number concentration, mass concentration, and specific surface concentration, as a function of aerodynamic diameters. Particles with a size below 2.5 µm (PM_2.5_) are very dangerous for the machine operator and environment [29]. In the present study, the fine particles (PM_10_) were collected as output parameters for the investigation.

**Figure 15:**
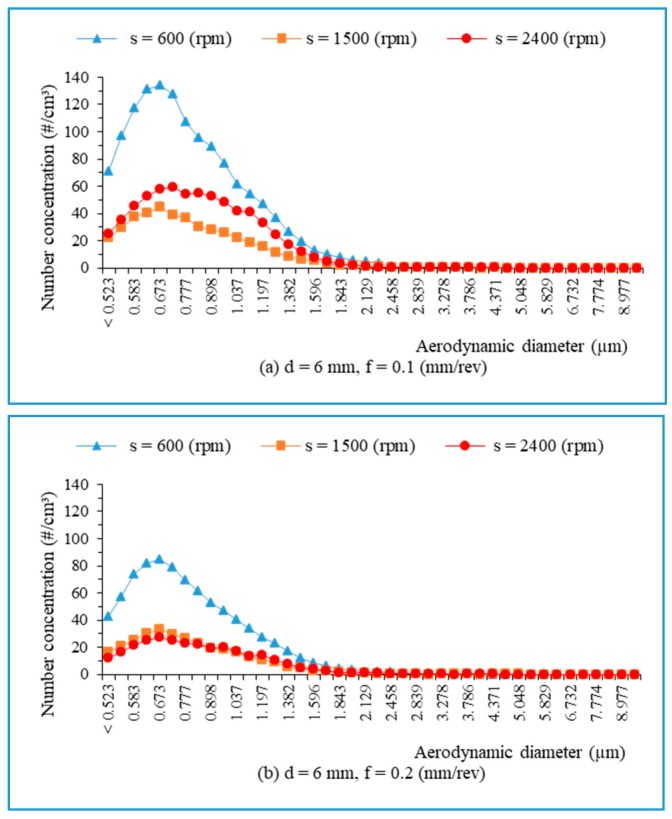
The number of fine particles versus aerodynamic diameters during drilling, with a drill of 6 mm: (**a**) f = 0.1 mm/rev; and (**b**) f = 0.2 mm/rev.

From the collected data, it is found that large quantities of fine particles with sizes smaller than 2.5 μm (PM_2.5_) are generated during dry drilling of hybrid biocomposite (approximately 98% of total fine dust). Figure 15 presents the number of fine particles in relation to aerodynamic diameters when drilling hybrid biocomposite, with a drill of 6 mm and various spindle speeds. It is also seen that most particles generated are of a size ranging from 0.5 μm to 1.5 μm, with a peak occurring at 0.673 μm. The observation indicates that the number of fine particles decreased with the feed rate increase (Figure 15a,b).

**Figure 16:**
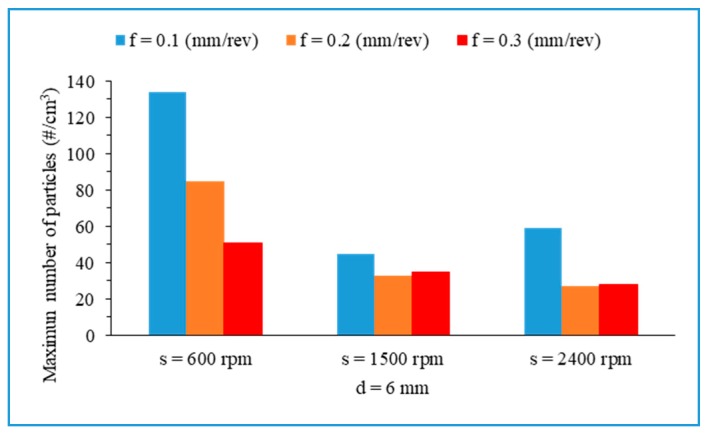
Peak value of number of fine particles related to particle size.

Figure 16 presents the maximum value of number of fine particles obtained during the drilling of hybrid biocomposite with different cutting parameters, with a drill bit diameter of 6 mm. According to the collected data, most of the maximum number concentration values were obtained with a size of 0.673 μm, except for one case (f = 0.1 mm/rev, s = 2400 rpm), a particle size of 0.723 μm has a peak value of 59 (#/cm^3^), followed by a size of 0.673 μm with 58 (#/cm^3^). Therefore, it can be deduced that the fine particle with a size of 0.673 μm achieved the greatest number concentration versus other sizes in the same cutting condition.

From Figure 16, it can be seen that the spindle speed increase results in a decrease in the maximum fine particle number concentration value. It may be inferred that the rise of the spindle speed may cause particles generated to be dispersed away from the machining area, thereby reducing the number of measured particles near the cutting zone.

**Figure 17:**
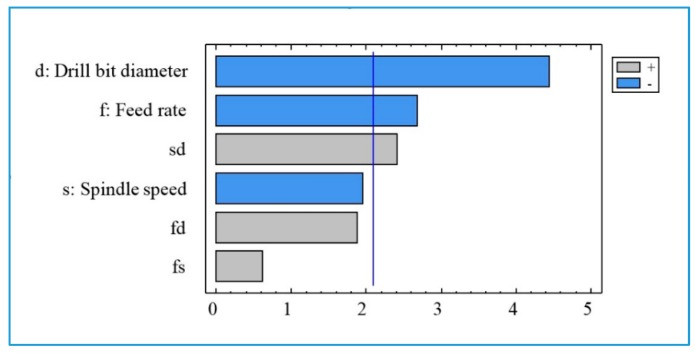
Pareto diagram for total number of fine particles (PM_10_).

The Pareto diagram (Figure 17) shows that the drill bit diameter (d) has the greatest effect on the fine particles (PM_10_), followed by the feed rate (f) and the interaction between the speed and the drill diameter (sd). The spindle speed (s) and interactions fs and fd have no statistical significance for fine particles emission. 

**Figure 18:**
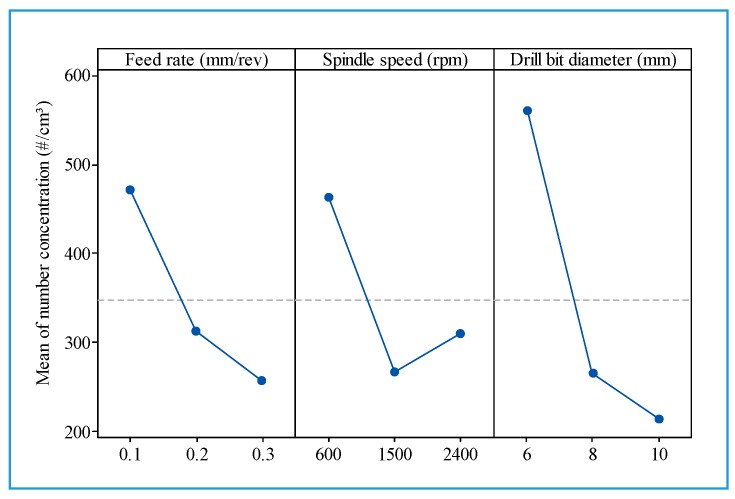
Main effect plot for fine particles emission.

**Figure 19:**
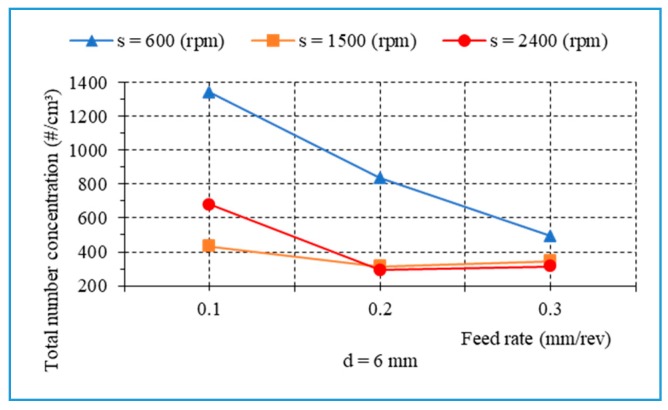
The number of fine particles versus the feed rate during drilling with different cutting parameters, d = 6 mm.

Figure 18 indicates that an increase in feed rate and/or drill bit diameter contributes to a reduction in the total number of fine particles. Figure 19 presents the total number concentration of fine particles in relation to the feed rate when drilling hybrid composite at different spindle speeds, with a drill of 6 mm. It is observed that the rise in feed rate leads to a decrease in the total number of fine particles. 

**Figure 20:**
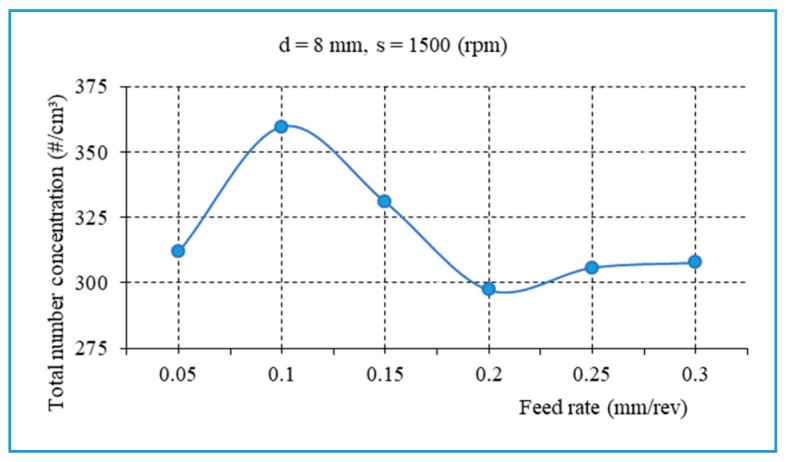
The fine particles related to the feed rate, with d = 8 mm, s = 1500 rpm.

**Figure 21:**
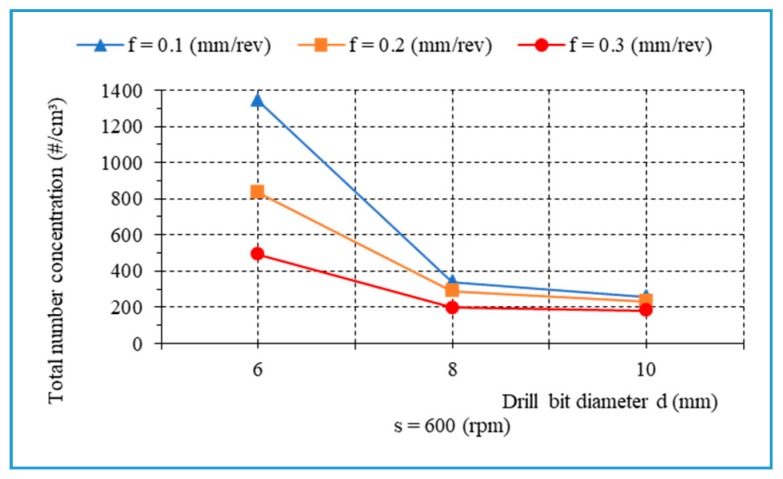
The number of fine particles related to drill bit diameter when drilling, with different feed rates, s = 600 rpm.

In order to better understand the influence of the feed rate on the fine dust generated, additional experiments were conducted with s = 1500 rpm, d = 8 mm, and f = 0.05 − 0.3 mm/rev (Figure 20). It is observed that the main trend of the total number of fine particles points to a slight decrease as the feed rate increase. Figure 21 presents the total number concentration of fine particles as a function of drill bit diameter during drilling of biocomposite with different feed rates, s = 600 rpm. It was found that a larger drill bit diameter contributes to a decrease in the total number concentration of fine particles.

#### 3.4.2. Ultrafine Particle (UFP) Emission

**Figure 22:**
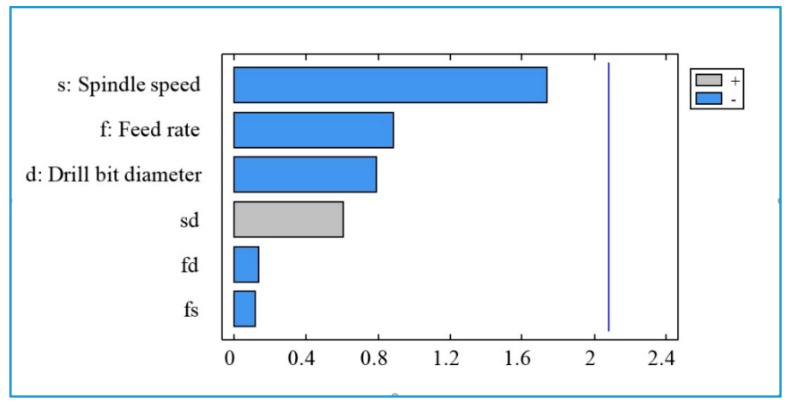
Pareto diagram for ultrafine particles emission.

Ultrafine particles with sizes ranging between 7 nm and 100 nm were used for the analysis presented in this sub-section. The Pareto diagram (Figure 22) indicates the influence of the tested factors and their interaction on the total number concentration of ultrafine particles during the drilling process. The tested factors and their interactions do not have statistically significant effects on ultrafine particles emitted at 95% confidence level. It was found that the spindle speed has a higher effect on ultrafine particles than do the feed rate and the drill’s diameter.

**Figure 23:**
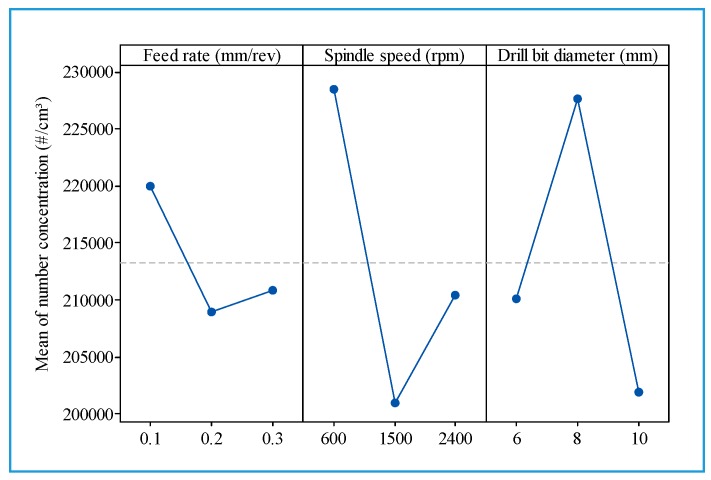
Main effect plot for total number concentration of ultrafine particles.

From Figure 23, it can be observed that the cutting conditions have an unclear influence on ultrafine particles emitted. Generally, the number concentration decreased with increasing spindle speed, drill bit diameter, and feed rate.

**Figure 24:**
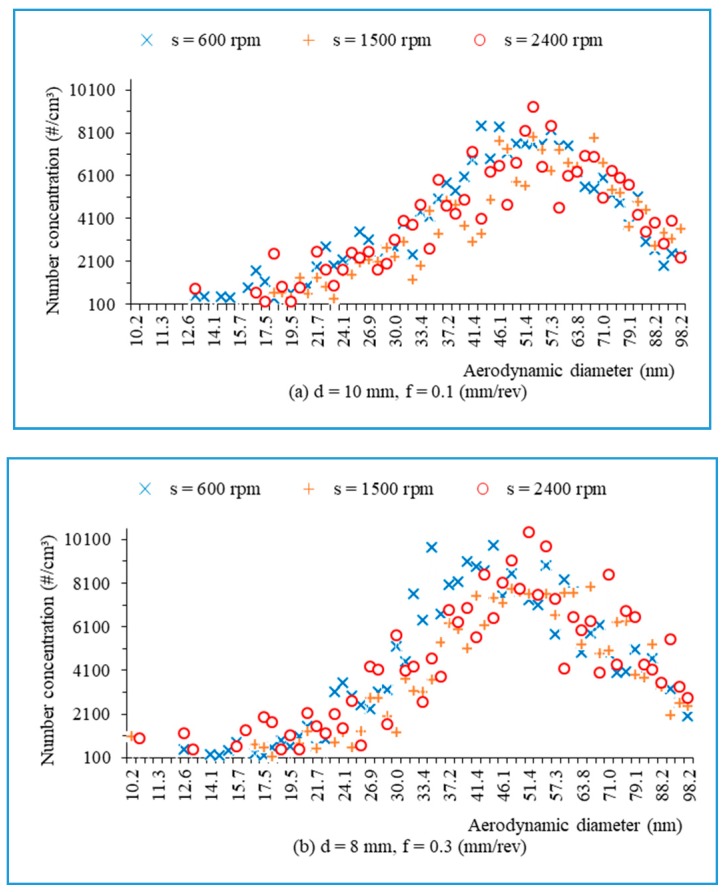
The number of ultrafine particles in relation to aerodynamic diameters when drilling with various machining parameters: (**a**) d = 10 mm, f = 0.1 mm/rev; (**b**) d = 8 mm, f = 0.3 mm/rev.

Figure 24 presents the number concentration of ultrafine particles versus the aerodynamic diameter during drilling of hybrid biocomposite at different spindle speeds, with f = 0.1(mm/rev), d = 10 mm (Figure 24a), and with f = 0.3 (mm/rev), d = 8 mm (Figure 24b). It is seen that most numbers of ultrafine particles have aerodynamic diameters ranging from 30 nm to 100 nm, with the greatest amount being around 50 nm.

**Figure 25:**
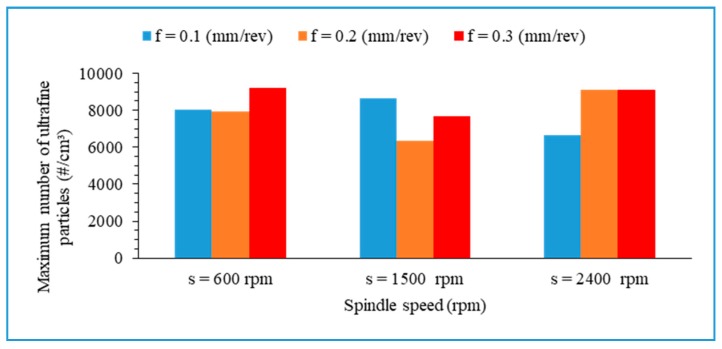
Relationship between maximum number concentration of ultrafine particles related to particle size and cutting parameters (drill: 6 mm).

Figure 25 shows the relationship between the peak value of number concentration of ultrafine particles and cutting parameters when drilling hybrid biocomposite with a drill of 6 mm. It was seen that the maximum value of the number concentration for ultrafine particles is not very sensitive to change in spindle speed and feed rate.

## 4. Conclusions

In this investigation, the effects of cutting parameters and drill bit diameter on machinability in the dry drilling of a new hybrid biocomposite were studied. Based on the experimental data and statistical technique employed, the following conclusions are drawn:

The machining conditions significantly influence the thrust force. The feed rate was found to have a higher effect on the thrust force than did the spindle speed and drill bit diameter. An increase in thrust force results from an increase in cutting parameters and drill bit diameter.

The specific cutting energy for the thrust force considered as a material property was investigated. This energy increased with an increase in spindle speed and decreased with an increase in feed rate and/or drill bit diameter.

The drill bit diameter was observed to have a greater impact on the surface roughness than did the cutting parameters. The surface roughness decreased with a decrease in the drill’s diameter, the spindle speed, and the feed rate. 

During drilling of this new composite material, both fine particles (PM_10_) and ultrafine particles (diameters ranging from 7–100 nm) were generated. The total number concentration of fine particles reached 1300 #/cm^3^, while the ultrafine particle generation reached 9000 #/cm^3^ depending on machining conditions used and on the particle size studied. The drill bit diameter and the feed rate have significant effects on the fine dust generation, while the spindle speed is not statistically significant. The total number concentration of fine particles decreased with an increase in feed rate, spindle speed, and/or drill bit diameter. More fine particles emitted had aerodynamic diameters less than 2.5 µm. The cutting parameters and the drill bit diameter did not show significant statistical effects on ultrafine particle generation during drilling of the hybrid composite at the 95% confidence level. Ultrafine particle generation was therefore difficult to predict.

## Figures and Tables

**Table 1:** Composition of hybrid biocomposite.

PP (%wt)	POE (%wt)	MAPP (%wt)	Biochar (%wt)	Miscanthus (%wt)
62	5	3	15	15

**Table 2:** Mechanical properties of hybrid biocomposite [38].

**Tensile strength (MPa)**	34
**Young’s modulus (MPa)**	3175
**Flexural strength (MPa)**	55
**Flexural modulus (MPa)**	2717
**Impact strength (J/m)**	140

**Table 3:** The design of experiments.

Factors	Level 1	Level 2	Level 3
f: Feed rate (mm/rev)	0.1	0.2	0.3
s: Spindle speed (rpm)	600	1500	2400
d: Drill bit diameter (mm)	6	8	10

**Table 4:** ANOVA for thrust force.

Source	Sum of Squares	D_f_	Mean Square	F-Ratio	*p*-Value
f: Feed rate (mm/rev)	9121.5	1	9121.5	104.28	0.0000
s: Spindle speed (rpm)	3949.53	1	3949.53	45.15	0.0000
d: Drill bit diameter (mm)	2801.01	1	2801.01	32.02	0.0000
Interaction f.s (mm/min)	126.945	1	126.945	1.45	0.2424
Interaction f.d (mm^2^/rev)	205.427	1	205.427	2.35	0.1411
Interaction s.d (rpm*mm)	1374.52	1	1374.52	15.71	0.0008
Total error	1749.41	20	87.4703		
Total (corr.)	19328.3	26			

**Table 5:** ANOVA for specific cutting energy.

Source	Sum of Squares	D_f_	Mean Square	F-Ratio	*p*-Value
f: Feed rate (mm/rev)	27,881.8	1	27,881.8	137.47	0.0000
s: Spindle speed (rpm)	7015.2	1	7015.2	34.59	0.0000
d: Drill bit diameter (mm)	4361.16	1	4361.16	21.50	0.0002
Interaction f.s (mm/min)	768.16	1	768.16	3.79	0.0658
Interaction f.d (mm^2^/rev)	388.855	1	388.855	1.92	0.1814
Interaction s.d (rpm*mm)	833.333	1	833.333	4.11	0.0562
Total error	4056.54	20	202.827		
Total (corr.)	45305.1	26			

**Table 6:** ANOVA for surface roughness R_a_.

Source	Sum of Squares	D_f_	Mean Square	F-Ratio	*p*-Value
f: Feed rate (mm/rev)	0.0418569	1	0.0418569	4.50	0.0465
s: Spindle speed (rpm)	0.388962	1	0.388962	41.86	0.0000
d: Drill bit diameter (mm)	0.710829	1	0.710829	76.50	0.0000
Interaction f.s (mm/min)	0.00213333	1	0.00213333	0.23	0.6370
Interaction f.d (mm^2^/rev)	0.006075	1	0.006075	0.65	0.4283
Interaction s.d (rpm*mm)	0.000133333	1	0.000133333	0.01	0.9058
Total error	0.185835	20	0.00929174		
Total (corr.)	1.33582	26			
